# Associations between Macrophyte Life Forms and Environmental and Morphometric Factors in a Large Sub-tropical Floodplain

**DOI:** 10.3389/fpls.2018.00195

**Published:** 2018-02-19

**Authors:** Berenice Schneider, Eduardo R. Cunha, Mercedes Marchese, Sidinei M. Thomaz

**Affiliations:** ^1^Instituto Nacional de Limnología (INALI-UNL-CONICET), Santa Fe, Argentina; ^2^Programa de Pós-Graduação em Ecologia de Ambientes Aquáticos Continentais, Universidade Estadual de Maringá, Maringá, Brazil; ^3^Facultad de Humanidades y Ciencias (FHUC-UNL), Santa Fe, Argentina

**Keywords:** aquatic plants, ecosystem processes, connectivity, hydrological period, floodplain, Middle Paraná River

## Abstract

Macrophyte assemblages are composed of species with different life forms and various ecological functions. Our aim was to investigate the potential environmental determinants of changes in the biomass of individual life forms and of the composition of the macrophyte assemblage in terms of life forms diversity. We sampled 23 waterbodies at low and high water levels in the Middle Paraná River floodplain. Macrophyte biomass samples were collected and classified in terms of life forms. We performed a redundancy analysis using the biomass of the various life forms to assess the importance of environmental variables to the composition of macrophyte life forms. Linear regressions were applied to investigate the environmental determinants of the biomasses of individual life forms. The degree of connectivity and the combination of depth, hydrology and nitrate were the main determinants of the composition in terms of life forms. The biomass of each individual life form was explained by different combinations of environmental variables, but the connectivity was the most important one. Our study shows that groups of species with similar life forms respond to environmental factors in particular ways, which might alter the biomass composition of life forms. Given that the ecosystem functioning depends on the functional characteristics of local communities, our findings about the relation between environmental changes and the community composition in terms of life forms (or functional composition) can be a helpful tool for predicting changes on ecosystem processes (such as nutrient cycling) against possible future scenarios.

## Introduction

The world's biodiversity is being lost at an unprecedented speed (Díaz et al., 2006) mainly as a consequence of human changes of the global environment (Chapin et al., 2000). These changes in biodiversity modify ecosystem processes and alter the resilience of ecosystems to environmental change (Chapin et al., 2000; Díaz et al., 2006; Cardinale et al., 2012) as sets of species with particular traits are replaced by other sets with different traits (Grime et al., 2000).

The importance of environmental regulation on the life forms of plants might be strong in aquatic ecosystems, where environmental conditions are commonly reported to be the major forces structuring macrophyte communities (Junk et al., 1989; Padial et al., 2009; Bornette and Puijalon, 2011; Powell et al., 2014; Schneider et al., 2015; De Wilde et al., 2016; Riera et al., 2017). In these ecosystems, different life forms (e.g., emergent, rooted submerged, and free-floating) use resources (e.g., light and nutrients) in specific ways and also differed in their response to environmental changes (Akasaka and Takamura, 2011; Netten et al., 2011; Alahuhta et al., 2013a). For example, while submerged plants are strongly dependent on under-water light (Sand-Jensen and Sondergaard, 1979; Chambers and Kalff, 1985; Zhang et al., 2012; Luhtala et al., 2017), free-floating plants have primacy in obtaining this resource (Lacoul and Freedman, 2006), but are more affected by nutrients availability in the water column (Henry-Silva et al., 2008; Giblin et al., 2014). On the contrary, emergent macrophytes are influenced by light availability only in the early stage of development and obtain nutrients from sediment, being expected as less dependent on water quality than other life forms (Akasaka et al., 2010; Akasaka and Takamura, 2011) (but see O'Hare et al., 2012; Alahuhta et al., 2013a,b; for contrasting results). However, changes in water quality can affect emergent macrophytes indirectly, for example, through organic matter sedimentation (Partanen et al., 2009).

In addition to limnological variables, the morphometric characteristics of the waterbodies greatly control the macrophyte biomass (Hudon et al., 2000) and distinctly affect life forms (Blanch et al., 1999; Van der Valk, 2005; Alahuhta and Heino, 2013; Alahuhta et al., 2013a). In river-floodplain systems, for example, water level variations directly influence emergent macrophytes biomass (Wetzel, 2001) and indirectly affect submerged plants through the reduction of underwater radiation (Chambers and Kalff, 1985). Wave action, measured indirectly by degree of wind exposure (fetch), can damage free floating and emergent plant tissues, or can influence submerged macrophytes by sedimentation and the resuspension process (Madsen et al., 2001; Zhang et al., 2014).

Similarly, disturbances caused by flow pulses are more likely to affect ungrounded or weakly grounded species, such as free-floating and submersed species, and favor those strongly fixed in the sediment, such as rooted floating-steamed plants (Junk and Piedade, 1993; Cronk and Fennessy, 2001). Such large-scale events also drive changes in terms of habitat suitability, leading to conditions that restrict some life forms, such as increasing depth for emergent species (Akasaka et al., 2010). In this sense, environmental disturbances at large spatial scales cause the substitution of macrophyte species and macrophyte life forms, which in turn change the macrophyte taxonomic and composition of life forms across the landscape (e.g., Neiff, 1979; Bini, 1996; Junk and Piedade, 1997; Morandeira and Kandus, 2016).

Disparities in terms of habitat use and interactions with environmental conditions among different life forms lead to different mosaics of macrophyte life forms at finer spatial scales (Fu et al., 2014). Because macrophytes are important components of aquatic ecosystems in terms of biomass production and habitat structuring and present particularities regarding the responses of life forms to environmental factors, understanding the relationships between life forms and environmental and morphometric factors might provide the stepping stone to improving the knowledge of ecosystem processes. However, most of the investigations in aquatic ecosystems using a functional approach have focused on the drivers of richness and composition of macrophyte life forms (Akasaka et al., 2010; Akasaka and Takamura, 2011; Netten et al., 2011; Alahuhta and Heino, 2013; Alahuhta et al., 2013a; Alahuhta, 2015; Morandeira and Kandus, 2016; Toyama and Akasaka, 2017, among others), but research on the relationship between the biomass (or primary productivity) of particular life forms and the changes in environmental factors is still scarce (Sand-Jensen and Sondergaard, 1979; Lampert and Sommer, 1997; Hudon et al., 2000; Stromberg and Merritt, 2015). Because the mechanisms by which diversity is expected to affect ecosystem functioning depend on the functional characteristics of local communities, assessing the relative importance of the abiotic variables to the biomass of particular life forms and to the life forms composition of the assemblages is key for comprehending the structure and dynamics of ecosystems.

Here, we built on previous investigations exploring the relationship of the taxonomic structure of macrophyte assemblages with environmental variables within a relatively unaltered floodplain (Schneider et al., 2015) and investigated the potential roles of environmental variables on changes in the biomass composition of different macrophyte life forms. Our aim was to identify the main environmental determinants of individual life forms of macrophytes and of the life form composition of the macrophyte assemblage. We hypothesize that particular life forms are differently correlated with limnological and morphometric factors and that these factors determine the composition of a macrophyte assemblages in terms of life forms. We expected the flood pulse to be among the most important environmental factors related to changes in the biomass of life forms because this factor is a major driver of changes in aquatic systems (Junk et al., 1989; Neiff, 1990).

## Materials and methods

### Study area

**Figure 1:**
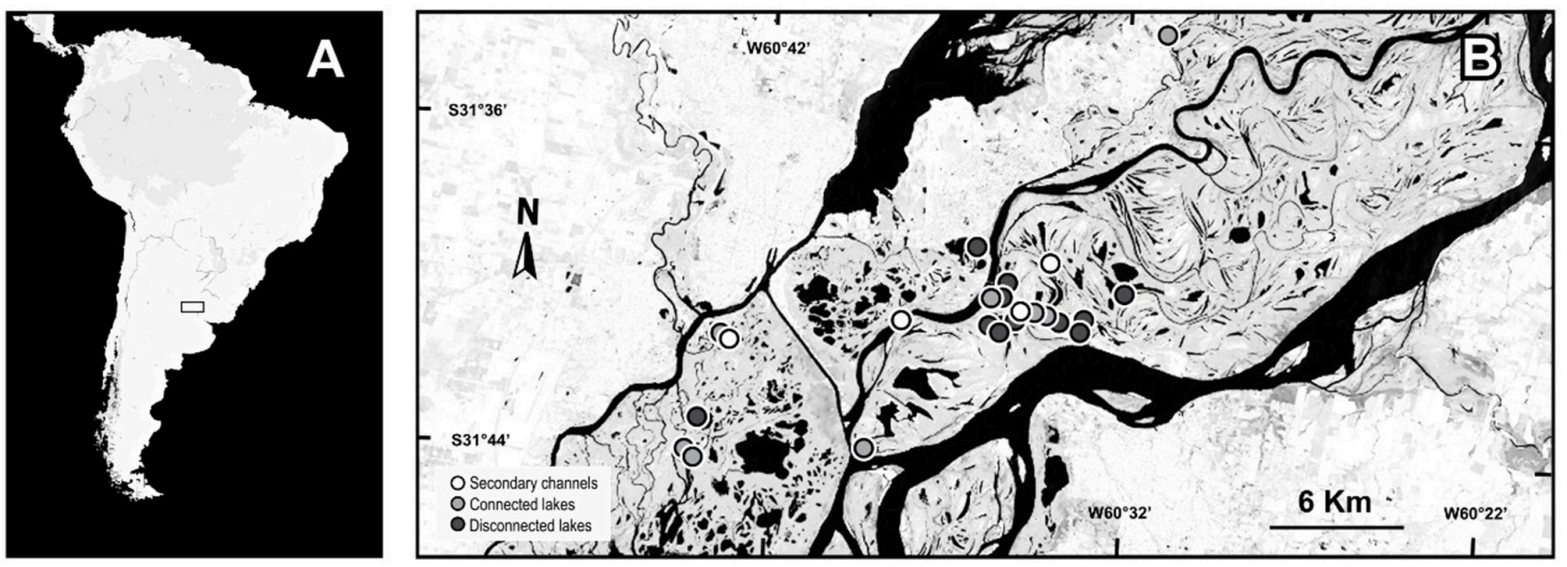
Map of the geographical location of the study area. **(A)** South America. **(B)** The Middle Paraná River with the sampling sites.

The floodplain of Middle Paraná River (Figure 1) is located between the confluence of this river with the Paraguay River (27°29′S; 58°50′W) and the city of Diamante (Argentina) (32°4′S; 60°32′3″W) (Iriondo and Paira, 2007). This stretch shows regular annual variations in water levels, with the wet and dry seasons lasting from January to June and from July to December, respectively. During the wet season, an increase in the water level can overflow the riverbed and inundate the whole floodplain (Drago, 2007). The combination of this variation with the complex geomorphology of the landscape ensures a mosaic of lotic and lentic water bodies that can be permanent or intermittent over the year. For example, some secondary channels remain connected to the riverbed throughout the year, whereas minor channels can cease their flow, beginning cyclical “lentification processes” (Drago et al., 2003). Such a complex riverine landscape supports a great diversity of species, which depends on the seasonal dynamic for maintaining the regional pool (Paira and Drago, 2007). Aquatic macrophytes are particularly diverse within these ecosystems (Neiff et al., 2014) and comprise an important portion of the system biomass (Sabattini and Lallana, 2007).

### Field sampling

The study was conducted in 23 waterbodies: eight connected lakes, 11 isolated lakes and four secondary channels of the Middle Paraná River floodplain. The connected lakes present a direct and permanent connection with the main river or secondary channels through a mouth, an erosion ditch levee or a short channel <1 km in length (Drago, 1981). In general, connected lakes present an in-flow connection with channels during the rising-channeled water phase until the river reaches the bankfull stage; and an out-flow connection during the falling-drainage water phase which starts when the river stage falls below the bankfull level, originating a flow from the lentic water bodies to the main and floodplain channels (Drago, 2007). Isolated lakes do not present any surface connection with the river and the inflow of river water occurs only during the peaks of inundation phases through the floodplain surface. The secondary channels sampled were permanent lotic water bodies with a constant flow throughout the year ensuring the annual lotic connectivity between the main channel and the floodplain (Drago et al., 2003). Different numbers of each environmental group were sampled to resemble the real availability of environments in the landscape. The samplings were performed during a dry (Oct 2012–Jan 2013) and a flood period (Aug-Sep 2013). The mean water level differed by 1 m between low- and high-water periods, but a marked flood pulse at the beginning of the high-water period ensured environmental differences between periods (see details in Supplementary Material 1). In low waters the minimum depth was of 24 cm in isolated lakes, 2.9 cm in connected lakes and 16.0 cm in secondary channels while the maximum depth was of 74.4 cm in isolated lakes, 55.4 cm in connected lakes and 79.5 cm in secondary channels. In high waters the minimum depths were 33, 7.3, and 116.6 cm in isolated lakes, connected lakes and in secondary channels, respectively; while the maximum values were 137, 193, and 210 cm in isolated lakes, connected lakes and secondary channels, respectively.

In each waterbody, the macrophyte biomass was sampled using a 0.25 m2 quadrat that was placed along transects perpendicular to the shoreline. The number of transects varied from 1 to 5 depending on the area of the waterbody (total of 86 transects). Transects length ranged between 1 m (e.g., in channels) and 40 m (e.g., in elongated lakes) depending on the macrophyte stand size. Within each transect, the number of quadrats varied from 1 to 13 according to the transect length (total of 289 quadrats). Distances between successive quadrats was kept constant within each transect but varied among transects. Despite of such a difference, the number of quadrats sampled in each transect was directly proportional to the stand size. This procedure takes into account habitat area and the size of the macrophyte stands, ensuring a representative inventory of aquatic flora. In addition, this procedure accounts for the zonation along depth gradients and the variation of spatial distribution in the margins, allowing the investigation of associations between macrophyte assemblages attributes and environmental variables. Inside each quadrat, the macrophytes were cut with scissors, removed from the bottom and kept in plastic bags. Rakes were used to collect submerged plants, and only the aboveground biomass was considered. In the laboratory, the macrophytes were washed to reduce their contents of periphyton and other debris and dried in an oven (60°C) to a constant weight (dry weight).

The macrophytes were identified to the maximal taxonomic resolution possible according to Cabrera (1968), Pott and Pott (2000), and other researchers (complete list in Supplementary Material 2), and the species nomenclature followed the standards of Missouri Botanical Garden's TROPICOS database (http://tropicos.org/). To describe different forms of exploiting resources, which might indicate different functions in an aquatic ecosystem, we separated the macrophytes into seven different life forms as proxies of functional groups: (i) *emergent* (Em) species, which have aerial stems and leaves growing above the water surface and use nutrients from the sediment [e.g., *Ludwigia peploides* (Kunth) P.H. Raven, *Polygonum punctatum* Elliott, *Thalia geniculata* L.]; (ii) *rooted floating-stemmed* (RFS) species, which acquire nutrients from the sediment and water and spread their stems over the water surface, with leaves emerging from the water column [e.g., *Eichhornia azurea* (Sw.) Kunth, *Paspalum repens* Bergius, *Hymenachne amplexicaulis* (Rudge) Nees]; (iii) *rooted floating-leaved* (RFL) species, which use nutrients from the sediment and have petioles that reach the water surface and floating leaf blades [e.g., *Nymphoides indica* (L.) Kuntze, *Victoria cruziana* Orb.]; (iv) *free floating* (FF) species, which have leaves above the water surface and submerged roots absorbing nutrients from the water column [e.g., *Pistia stratiotes* L., *Salvinia biloba* Raddi, *Eichhornia crassipes* (Mart.) Solms]; (v) *free submerged* (FS) species, which remain freely beneath the water surface or attached to other macrophytes or structures and use the nutrients and light available in the water column (e.g., *Ceratophyllum demersum* L.); (vi) *rooted submerged* (RS) species, which have stems and leaves underwater and are rooted in the sediment, from where they uptake most of their required nutrients (e.g., *Potamogeton pusillus* L., *Cabomba caroliniana* A. Gray); and (vii) *epiphytic* (Ep) species, which have stems and leaves above the water surface and usually have roots above free-floating plants, allowing them to obtain nutrients from superficial water and from the debris accumulated over their substrate [e.g., *Oxycarium cubense* (Poepp. and Kunth) Palla *cubense*].

Environmental parameters were measured at different scales (Table 1) encompassing the degree of connectivity with the river main channel, physical and chemical variables and morphometric features (depth, littoral slope, and fetch). The degree of connectivity with the river main channel was considered a categorical variable and it is highest in secondary channels (2), intermediate in the connected lakes (1) and lowest in the isolated lakes (0). In each transect, we measured the conductivity and pH with portable water checkers (Conductivity meter HI 9,033 Hanna® and pH meter HI 8,424 Hanna® respectively). The Secchi depth in each transect was determined to obtain an estimate of the light attenuation coefficient (according to Padial and Thomaz, 2008). The depth was measured in the center point of each quadrat. The littoral slope was calculated for each transect using depth measurements and distances between the quadrats to the shoreline. For each transect, we also calculated the maximum distance of open water in a straight line from the macrophytes stand to the furthest point to the shoreline or an island and used this distance as a surrogate of the fetch. For each waterbody, we determined the total phosphorus, nitrate and ammonium contents (with HACH; Greenberg et al., 1985). For this purpose, we sampled water at the sub-surface in the border between the macrophyte stand and the limnetic region of the waterbody. We also estimated the organic matter content of the sediment by grabbing a sediment sample at a depth of 60 cm (± 10 cm) from areas colonized by macrophytes and igniting the sample in a muffle furnace at 500°C for 3 h.

**Table 1:** Summary of the response and explanatory variables sampled in the study.

**Variable**	**Abbreviation**	**Sampling scale**	**Rescaling for analysis**	**Min**.	**Q1**	**Med**.	**Q3**	**Max**
**BIOMASS OF MACROPHYTE LIFE FORMS**								
Emergent (g)	Em	Q	Qs' sum for each T, and mean T per W	0.0	57.1	226.3	677.5	3325.5
Free floating (g)	FF	Q	Qs' sum for each T, and mean T per W	0.0	9.5	159.1	264.4	1391.8
Epiphytic (g)	Ep	Q	Qs' sum for each T, and mean T per W	0.0	0.0	0.0	0.5	56.2
Rooted submerged (g)	RS	Q	Qs' sum for each T, and mean T per W	0.0	0.0	0.0	0.8	136.7
Free submerged (g)	FS	Q	Qs' sum for each T, and mean T per W	0.0	0.0	0.0	4.0	414.0
Rooted floating-leaved (g)	RFL	Q	Qs' sum for each T, and mean T per W	0.0	0.0	0.4	9.4	459.7
Rooted floating-stemmed (g)	RFS	Q	Qs' sum for each T, and mean T per W	0.0	0.0	12.0	75.2	682.5
**ENVIRONMENTAL DESCRIPTORS**								
Hydrologic period	Hyd	W	None	0.0	0.0	0.0	1.0	1.0
Degree of connectivity	Conec	W	None	0.0	0.0	1.0	1.0	2.0
Fetch (Km)	Fetch	T	Ts' mean per W	0.0	0.1	0.2	0.5	2.6
Depth of water column (cm)	Depth	Q	Qs' mean for each T, and mean T per W	2.9	31.3	51.2	77.6	210.0
Slope of littoral zone	Slope	T	Ts' mean per W	0.0	0.0	0.1	0.1	0.3
Estimates of light attenuation coefficient	k_est_	T	Ts' mean per W	0.0	0.1	0.1	0.2	0.4
Conductivity (μS cm^−1^)	Cond	T	Ts' mean per W	37.7	85.3	105.8	125.3	230.0
Nitrate (ppm)	Nit	W	None	0.1	0.2	0.7	1.6	6.3
Ammonium (ppm)	Amo	W	None	0.1	0.2	0.3	0.4	0.7
Phosphorus (ppm)	Pho	W	None	0.0	0.1	0.2	0.6	1.5
% organic matter (%)	OM	W	None	0.9	4.8	8.4	12.3	26.1
# sampled quadrats	#Quad	T	Ts' mean per W	1.0	6.0	8.0	9.0	13.0

*The initials Q, T, and W indicate whether the samples were taken from a quadrat, a transect and a waterbody, respectively. “Rescaling for analysis” describes the operations performed to rescale the data to the waterbody scale (“Qs' sum for each T, and mean T per W” indicate that the data obtained in the quadrats of each transect were summed, and the sums obtained for each transect were then averaged in each waterbody; “Qs' mean for each T, and mean T per W” indicate that the data were averaged in each transect, and the averages obtained for each transect were then averaged in each waterbody”; “Ts' mean per W” indicate that the data (in this case, obtained at the transect scale) were averaged in each waterbody; and “None” indicate that no transformation was needed given that the data were measured at the waterbody scale)*.

### Data processing

Biomass of each species was obtained separately to ensure that the macrophyte biomass could be considered by each life form or total plant material. Prior to the analyses, all response and explanatory variables were scaled up to the water body level (details in Table 1). To achieve that, the data obtained in the quadrats of each transect were summed and the sums obtained for each transect were averaged in each waterbody; the data obtained at the transect scale were averaged in each waterbody; while the data measured at the waterbody scale was not transformed. Because the samples were systematically collected across transects perpendicular to the shoreline, we could capture the variation in macrophyte zonation along the littoral and assess the overall relationships between macrophyte biomass and environmental conditions. This approach might also account for potential pseudo-replication (e.g., dependency of quadrats within transects) while preserving valid relationships between the macrophyte biomasses and explanatory variables. Thus, the individual waterbodies represented our sample units.

### Statistical analysis

To assess the importance of environmental factors to variations in the composition of macrophyte life forms in terms of biomass (for the sake of simplicity, hereafter denoted “composition of macrophyte life forms”), we employed a Redundancy Analysis (RDA). We used a modified Hellinger transformation to account for the unimodal responses of macrophyte life forms to environmental gradients and reduce the influence of abundant life groups in the ordination (Legendre and Gallagher, 2001). The adaptation of the Hellinger transformation considered altering the square root by log in the transformation routine to more strongly downweigh dominant life groups and allow the expression of non-dominant species (P. Legendre and D. Borcard, pers. comm.). Some environmental variables were then root-transformed to linearize their relationships with explanatory variables and improve the fit of the data to normal distributions. Model building to provide the best subset of environmental variables explaining the composition of macrophyte life forms was performed by a forward stepwise procedure. Double stop criteria were considered (significant level for variable inclusion and R2_adj_ of the full model) to avoid type I errors (Peres-Neto et al., 2006; Blanchet et al., 2008).

We also investigated the environmental variables that best explain the biomass of each particular life form of macrophytes. To achieve this goal, we used linear regressions, which parallel RDA for univariate approaches (Blanchet et al., 2008). For the sake of simplicity and coherence with protocols used for RDA, we applied similar transformations and performed the same procedure to select variables that significantly influence the biomass of individual life forms. All of the analyses were performed in the R environment (v. 3.2.3) using the *vegan, stats, packfor* and *ad4* packages.

## Results

A total of 55 macrophyte taxa were registered (Supplementary Material 3). Of these, 27 were classified as E, one Ep, nine FF, three FS, five RS, three RFL, and seven RFS.

**Figure 2:**
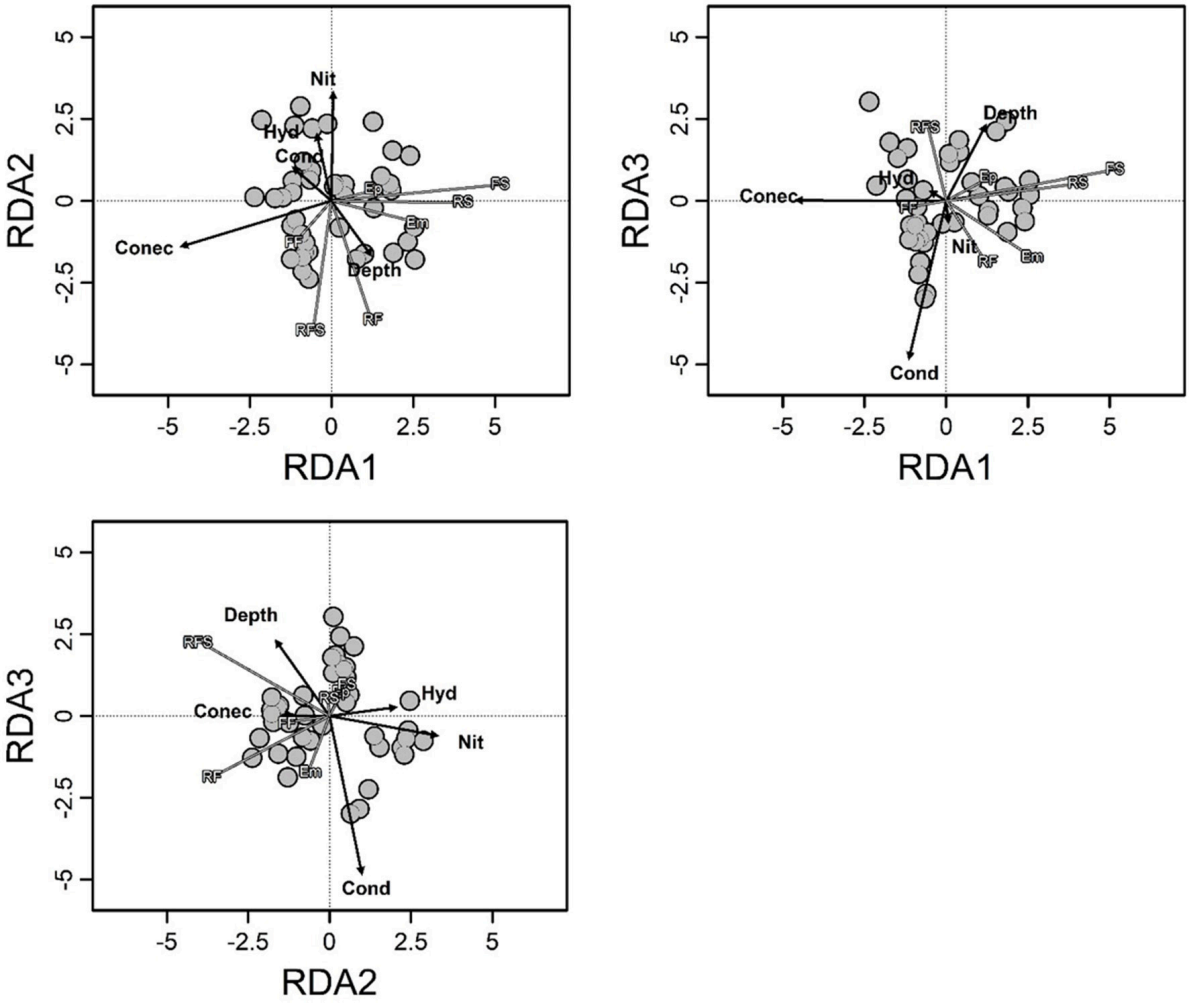
Triplot of redundancy analysis showing the biomass composition of the macrophyte life forms in relation to the environmental variables. Arrows indicate the direction and relative importance of the environmental variables. Hyd, hydrological period; Conec, degree of connectivity; Cond, conductivity; Depth, fourth root of depth; Nit, fourth root of nitrate; Em, emergent; FF, free floating; Ep, epiphytic; RS, rooted submerged; FS, free submerged; RFL, rooted floating-leaved; RFS, rooted floating-stemmed.

Environmental variables explained 30% of the total variation (R2_adj_ = 0.30; *F* = 4.34; *p* < 0.001) in composition of macrophyte life forms, and the degree of connectivity, depth, nitrate, conductivity and hydrologic period had significant contributions (Supplementary Material 4 and Figure 2). Among these variables, the most important changes in the composition of macrophyte life forms were associated with the degree of connectivity and the combination of depth, hydrologic period and nitrate (with RDA1 and RDA2 representing 83% of the total variance explained).

Most environmental variables that were correlated with changes in the biomass composition were also correlated with the biomass of at least one of the individual life forms. For these analyses, the explanatory power of the environmental variables varied between 0 and 48% (R2_adj_), suggesting that environmental variables can play a potential role in regulating the biomass of life forms at different strengths (Table 2).

**Table 2:** Results of regression analyses performed to explain the biomass of different macrophyte life forms.

**Macrophyte life form**	**$R_{\text{adj}}^{2}$**	**Standardized regression coefficient**									
		**Coef. (SE) and *p*-value**	**Hyd**	**Conec**	**Fetch**	**Slope**	**Depth**	**k_est_**	**Cond**	**Nit**	**Amo**
Emergent (Em)	0.45	β	-	−1.53 (0.66)	−2.42 (0.66)	-	-	-	-	-	-
		*p*	-	0.02	0.001	-	-	-	-	-	-
Epiphyte (Ep)	0.00	β	-	-	-	-	-	-	-	-	-
		*p*	-	-	-	-	-	-	-	-	-
Free floating (FF)	0.08	β	−1.84 (0.85)	-	-	-	-	-	-	-	-
		*p*	0.04	-	-	-	-	-	-	-	-
Free submerged (FS)	0.48	β	-	−5.46 (0.89)	-	-	-	-	-	-	-
		*p*	-	>0.001	-	-	-	-	-	-	-
Rooted submerged (RS)	0.28	β	-	−4.01 (0.98)	-	-	-	-	-	-	-
		*p*	-	>0.001	-	-	-	-	-	-	-
Rooted floating-leaved (RFL)	0.26	β	-	-	-	-	-	−3.44 (1.17)	-	−2.69 (1.19)	2.56 (1.14)
		*p*	-	-	-	-	-	0.005	-	0.03	0.03
Rooted floating-stemmed (RFS)	0.31	β	-	-	-	-	3.6 (1.11)	-	-	−4.06 (1.11)	-
		*p*	-	-	-	-	0.002	-	-	>0.001	-

*Environmental variables: hydrological period (Hyd), degree of connectivity (Conec), fetch (Fetch), slope (Slope), depth (Depth), estimated light attenuation coefficient (k_est_), conductivity (Cond), nitrate (Nit), and ammonium (Amo)*.

Consistent with the RDA results, the combinations of environmental variables explaining the variation in macrophyte biomass were markedly distinct among the individual life forms, with the exception of the degree of connectivity, which partially explained the variation in most cases (Table 2). The Em life form was negatively associated with fetch and connectivity. No variables were correlated with the changes in the biomass of the Ep. The biomass of FF plants was negatively related with the hydrological period. Both biomass of RS and FS life forms was negatively associated with the degree of connectivity. The changes in the biomass of RFL macrophytes were positively associated with ammonium but negatively related with k_est_ and nitrate. For RFS life form, plant biomass was positively associated with depth and was negatively related to nitrate.

## Discussion

Studies using the functional approach in the field of community ecology have usually focused on the relationships between abiotic factors and the richness and composition of macrophyte growth forms (e.g., Akasaka et al., 2010; Alahuhta and Heino, 2013; Alahuhta et al., 2013a; Alahuhta, 2015). Nevertheless, a more basic understandings of the potential effects of environmental variables on the biomass of different life forms (used as surrogates of functional groups) has generally been underestimated (Hudon et al., 2000). Our main results suggest that different morphometric, chemical and hydrological variables explain the variation in macrophyte life forms biomass and thus lead to changes in the life form composition across environmental gradients. In view of these results, our hypothesis that particular life forms are differently correlated with limnological and morphometric factors was not rejected. In addition to between-group particularities, our approach showed that the degree of connectivity between the waterbody and the riverbed (a morphometric variable) was consistently related to the biomass of most life forms and with the composition of macrophyte life forms. This result reinforces the major role of exchanges of water between the main river channel and floodplain waterbodies in regulating the macrophyte assemblages in river-floodplain ecosystems, as previously shown in studies that employed a taxonomic perspective (Junk et al., 1989; Neiff, 1990; Padial et al., 2009).

Our study revealed that the most important factors affecting the biomass and composition of macrophyte life forms are related to the morphometry of aquatic ecosystems. Morphometric factors have previously been identified as important determinants of the taxonomic structure and life forms diversity of macrophyte assemblages in many freshwater ecosystems (Sabattini and Lallana, 2007; Akasaka et al., 2010; Alahuhta et al., 2013a; Kissoon et al., 2013; Azzella et al., 2014; Alahuhta, 2015; Schneider et al., 2015). In particular, the connectivity and water levels are the main determinants of the composition of the assemblage in river floodplain ecosystems (e.g., Padial et al., 2009; Schneider et al., 2015; Evtimova and Donohue, 2016; Toth, 2017). Our results add information supporting the notion that not only the taxonomic but also the life form composition might be related to morphometry (in our specific case, connectivity, fetch, and depth).

Some variables that were found to be related with the biomass of life forms have previously been related to taxonomic attributes (e.g., Murphy et al., 2003; Schneider et al., 2015; Wang et al., 2016; Riera et al., 2017), which suggests that changes in the taxonomic structure can primarily result from the responses of life forms to environmental changes. For example, we found that changes in the biomasses of macrophyte life forms were strongly explained by the degree of connectivity, which was negatively related with three (Em, RS, and FS) out of seven life forms. Connected floodplain waterbodies are exposed to frequent water level fluctuations. This situation leads to a high instability of habitats, which may affect macrophyte life forms in different ways (Partanen et al., 2006; Neiff et al., 2008; Zhang et al., 2015; Wang et al., 2016). For instance, changes of water depth related to water level fluctuations could be catastrophic for RS (devoid of cuticle and supporting tissues) when exposed to air conditions or to very large floods (Sousa et al., 2010). Conversely, Em could be affected if they remain completely submerged for long periods. Furthermore, water flow (also associated with more connected waterbodies) (Bornette et al., 1998; Madsen et al., 2001) directly reduces submerged macrophyte biomass because of the strong mechanical strain and damage cause on plant tissues (Chambers et al., 1991; Madsen et al., 2001; Zhang et al., 2014). Particle resuspension induced by water movements can limit light penetration in the water and subsequently plant growth (Madsen et al., 2001). In general, submerged and Em macrophyte species lack traits to reduce the hydrodynamic forces or to increase mechanical resistance to breaking and uprooting, therefore are expected to be highly sensitive to water flow (Chambers et al., 1991; Madsen et al., 2001; Zhang et al., 2014). This relates to some particular characteristics of RS and FS, which generally lack supporting tissues (Sculthorpe, 1967) and are highly affected by flow, and many Em species, such as *Sagittaria montevidensis* Cham. and Schltdl. or *Alternanthera philoxeroides* (Mart.) Griseb, which have surficial root systems being susceptible to be uprooted or broken by water current (Sculthorpe, 1967). These features are likely to explain the negative correlation of RS, FS, and Em macrophytes with the degree of connectivity (see Table 1). Thus, the limiting conditions for species might depend more on the functional features of the species (represented by macrophyte life forms in our work) than on the species identity itself. As a result, the composition of macrophyte assemblages as a whole could respond to connectivity with an increasing biomass of RS and Em species in less-connected habitats, as was found in this study (see Figure 2).

The Em life form was also negatively affected by the fetch (a surrogate of wind exposure), another morphometric variable that regulates the distribution of freshwater macrophytes (Azza et al., 2007) (see Table 2). Em species can be negatively and directly related with fetch due to the damage caused by wind or waves or indirectly related to sediment scouring and uprooting plants (Azza et al., 2007; Bouma et al., 2009; Bal et al., 2011) or to limiting seedling establishment (Bouma et al., 2009). These negative effects on Em plants might be enhanced because these plants mainly colonize the shallowest portions of littoral zones where wave effects tend to be higher (Sculthorpe, 1967), likely explaining the negative relationships between fetch and Em species found in our work.

The biomass of the FF life form was negatively related with the hydrological period. During the flood pulses, FF plants are carried downstream by flow from floodplain lakes toward the main river (Sabattini and Lallana, 2007). This situation could drastically reduce the FF biomass within the floodplain habitats during high water phases, explaining the negative relation we found. However, the explanatory power of this variation was very low.

The RFS life form was found to be positively related with depth. The biomass and net primary productivity of many RFS species increase with increasing water level and, therefore, depth (Junk and Piedade, 1993). In the Middle and in other stretches of the Paraná River, the RFS life form generally colonizes river's margins (Neiff, 1986) which used to be deeper than lakes. Furthermore, the positive association with depth could be due to the fact that these plants tend to increase in biomass mostly by growing/expanding in the horizontal directions, easily reaching the deep areas of waterbodies (Junk and Piedade, 1997).

The biomass of the RFL life form was found to be positively associated with ammonium. This ion is the most easily absorbed nitrogen source for plants (James et al., 2004; Jampeetong and Brix, 2009). In addition, in the Middle and Lower Paraná basins, nitrogen is more limiting than phosphorus for plant growth (Carignan and Neiff, 1992; Bonetto et al., 1994). Thus, the positive correlation might indicate that habitats with a higher concentration of ammonium should promote RFL growth. The physical factor k_est_, which is related to water transparency, was negatively correlated with the RFL life form. Light availability has been demonstrated to stimulate both seed germination and seedling development of RFL plants (Sculthorpe, 1967; Smits et al., 1990; Huang et al., 2014). Thus, this negative association could be related with a lower rate of seed germination or a lower photosynthetic activity of RFL plant seedlings in more turbid habitats (Sculthorpe, 1967), since sufficient light is an important condition for both processes.

Interestingly, both the biomasses of RFL and RFS were negatively related to the nitrate level. Many soluble inorganic nutrients (such as nitrate) are removed by macrophytes from the water column by direct uptake (Greenway, 2007). Therefore, many species that can efficiently accumulate biomass might increase the nitrate uptake (James et al., 2005; Weisner and Thiere, 2010) and accumulate nitrogen in their biomass (Barbieri et al., 1984; Nogueira and Esteves, 1993), which in turn reduces the nitrate availability in the waterbodies.

## Conclusions

In summary, we found that the biomasses of macrophyte life forms respond differently to environmental changes, which might alter the biomass composition in terms of life forms. Among a set of abiotic factors, the degree of connectivity of the environments with the river along with other morphometric variables, such as depth, are the main determinants of the biomass of macrophyte life forms. Thus, not only species (which is the focus of more traditional taxonomic approaches) but also groups of species with similar life forms (functional approach, used in our work) respond to environmental factors in predicted ways. Thus, our results suggest that in river floodplain systems, the morphometric and hydrological variables are the primary determinants of the ecological processes (e.g., species sorting) that shaped macrophyte communities in terms of life forms. Given that the functioning of ecosystems depends on the functional characteristics of local communities, the link between environmental variables and the biomass of macrophyte life forms, such as those investigated in this study, can be a helpful tool for predicting the effects of environmental changes on ecosystem processes (such as nutrient cycling) against possible future scenarios.

## Author contributions

BS, EC, MM, and ST: designed the research; BS: collected the data; EC: performed the statistical analysis and BS, EC, MM, and ST: wrote the manuscript, commented on and approved the final draft.

### Conflict of interest statement

The authors declare that the research was conducted in the absence of any commercial or financial relationships that could be construed as a potential conflict of interest.
