# Supplementary material for: Associations between Macrophyte Life Forms and Environmental and Morphometric Factors in a Large Sub-tropical Floodplain
Source: Front Plant Sci. 2018 Feb 19;9:195. doi: 10.3389/fpls.2018.00195 (PMC5826054; doi:10.3389/fpls.2018.00195)
Supplement: Supplementary file 1 [file SupplementaryMaterial1.pdf]

Supplementary Material 1

Article — **Associations between macrophyte life forms and environmental and morphometric factors in a large sub-tropical floodplain**

List of Authors — Berenice Schneider*, Eduardo Ribeiro Cunha, Mercedes Marchese and Sidinei Magela Thomaz

***Correspondence:** Berenice Schneider: bereschneider@gmail.com

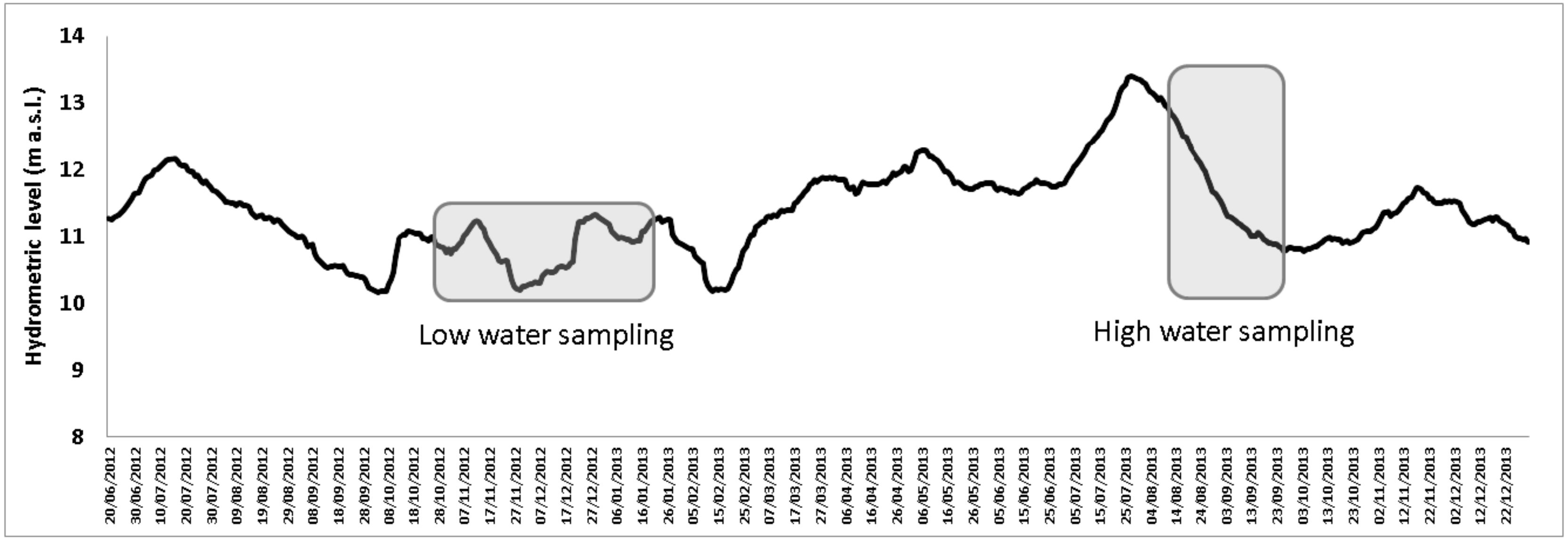

Supplementary Material 1 Hydrometric level in meters above sea level (m a.s.l.) during the two samplings periods.
