# Supplementary material for: Associations between Macrophyte Life Forms and Environmental and Morphometric Factors in a Large Sub-tropical Floodplain
Source: Front Plant Sci. 2018 Feb 19;9:195. doi: 10.3389/fpls.2018.00195 (PMC5826054; doi:10.3389/fpls.2018.00195)
Supplement: Supplementary file 2 [file SupplementaryMaterial2.pdf]

Supplementary Material 2

Article — **Associations between macrophyte life forms and environmental and morphometric factors in a large sub-tropical floodplain**

List of Authors — Berenice Schneider*, Eduardo Ribeiro Cunha, Mercedes Marchese and Sidinei Magela Thomaz

***Correspondence:** Berenice Schneider: bereschneider@gmail.com

Supplementary Material 2 Identification of macrophyte species.

All specimens of macrophyte species collected during field samples was transported to the Instituto Nacional de Limnología (INALI-UNL-CONICET). Plants were identified according to Cabrera (1968), Burkart (1969), Burkart (1974), Burkart (1979), Burkart (1987), Pott & Pott (2000) and Burkart & Bacigalupo (2005).

References

Burkart, A., 1969. Flora Ilustrada de Entre Ríos (Argentina), Tomo VI. Parte II. Colección Científica INTA, Buenos Aires.

Burkart, A., 1974. Flora Ilustrada de Entre Ríos (Argentina), Tomo VI. Parte VI. Colección Científica INTA, Buenos Aires.

Burkart, A., 1979. Flora Ilustrada de Entre Ríos (Argentina), Tomo VI. Parte V. Colección Científica INTA, Buenos Aires.

Burkart, A., 1987. Flora Ilustrada de Entre Ríos (Argentina), Tomo V. Parte III. Colección Científica INTA, Buenos Aires.

Burkart, A. & Bacigalupo, N.M., 2005. Flora Ilustrada de Entre Ríos (Argentina), Tomo IV. Parte IV. Colección Científica INTA, Buenos Aires.

Cabrera, A. L., 1968. Flora de la Provincia de Buenos Aires. Colección Científica INTA, Buenos Aires.

Pott, V.J. & Pott, A., 2000. Plantas aquáticas do Pantanal. Embrapa. Centro de Pesquisa Agropecuárica do Pantanal. Corumbá. EMBRAPA. Brazil.
