# Supplementary material for: Associations between Macrophyte Life Forms and Environmental and Morphometric Factors in a Large Sub-tropical Floodplain
Source: Front Plant Sci. 2018 Feb 19;9:195. doi: 10.3389/fpls.2018.00195 (PMC5826054; doi:10.3389/fpls.2018.00195)
Supplement: Supplementary file 3 [file SupplementaryMaterial3.pdf]

Supplementary Material 3

Article — **Associations between macrophyte life forms and environmental and morphometric factors in a large sub-tropical floodplain**

List of Authors — Berenice Schneider*, Eduardo Ribeiro Cunha, Mercedes Marchese and Sidinei Magela Thomaz

***Correspondence:** Berenice Schneider: bereschneider@gmail.com

Supplementary Material 3 List of macrophyte taxa registered with its respective life forms, averaged biomass during the low water (LW) and the high water (HW) samplings, and the number of waterbodies in which each taxa was registered during the low water (LW) and the high water (HW) periods.

Macrophyte taxa	Life forms	Averaged biomass (g/m²)		N° of waterbodies	
		LW	HW	LW	HW
Acmella decumbens (Sm.) R.K. Jansen var. decumbens	E	94,4	0	1	0
Alternanthera philoxeroides (Mart.) Griseb. f. *philoxeroides*	E	14,0	7,2	3	8
Ambrosia elatior L.	E	7,1	0	1	0
Azolla sp.	FF	25,5	7,1	17	16
Bidens laevis (L.) Britton, Stern & Poggenb.	E	32,0	23,0	4	4
Cabomba caroliniana A. Gray var. *caroliniana*	RS	7,5	7,3	4	4
Ceratophyllum demersum L.	FS	64,4	15,7	5	5
Commelina diffusa Burm. f. diffusa	E	4,0	0	1	0
Cyperus imbricatus Retz.	E	18,1	0	2	0
Cyperus sp.	E	10,9	0	4	0
Echinochloa polystachya (Kunth) Hitchc. var. *polystachya*	RFS	167,2	4,4	3	1
Echinochloa sp.	E	20,4	1,2	4	1
Eclipta prostrata (L.) L.	E	7,5	0	2	0
Egeria sp.	RS	23,9	2,6	5	3
Eichhornia azurea (Sw.) Kunth	RFS	87,6	54,0	8	5
Eichhornia crassipes (Mart.) Solms	FF	108,0	180,6	15	8
Eleocharis sp.	E	8,6	11,1	6	4
Elodea callitrichoides (Rich.) Casp.	RS	22,9	0	1	0
Enydra anagallis Gardner	E	23,7	12,1	12	8
Eryngium divaricatum Hook. & Arn.	E	3,4	0	3	0

Supplementary Material 3 (continuation) List of macrophyte taxa registered with its respective life forms, averaged biomass during the low water (LW) and the high water (HW) samplings, and the number of waterbodies in which each taxa was registered during the low water (LW) and the high water (HW) periods.

Macrophyte taxa	Life forms	Averaged biomass (m²)		N° of waterbodies	
		LW	HW	LW	HW
Hydrocotyle ranunculoides L. f.	RFS	8,2	9,5	10	7
Hymenachne amplexicaulis (Rudge) Nees	RFS	49,0	0	5	0
Lemna minuta Kunth	FF	0,2	0,1	10	3
Limnobium laevigatum (Humb. & Bonpl. ex Willd.) Heine	FF	22,6	20,5	14	12
Ludwigia peploides (Kunth) P.H. Raven subsp. *peploides*	E	253,7	61,3	18	19
Myriophyllum aquaticum (Vell.) Verdc.	E	47,8	21,1	10	14
Nitella sp.	RS	0,8	0	1	0
Nymphoides indica (L.) Kuntze	RFL	36,0	5,8	10	10
Oplismenopsis najada (Hack. & Arechav.) Parodi	E	62,9	28,7	4	9
Oxycaryum cubense (Poepp. & Kunth) Palla f. *cubense*	Ep	8,7	11,8	7	4
Panicum elephantipes Nees ex Trin.	RFS	73,2	116,0	3	2
Paspalum repens Bergius	RFS	81,5	126,3	7	10
Pistia stratiotes L.	FF	1,4	2,8	9	7
Polygonum acuminatum Kunth	E	21,1	0	1	0
Polygonum ferrugineum Wedd.	RFS	315,2	46,0	1	1
Polygonum hydropiperoides Michx. var. *hydropiperoides*	E	13,8	0	2	0
Polygonum lapathifolium L.	E	18,2	0	3	0
Polygonum paraguayense Wedd.	E	138,8	0	1	0
Polygonum punctatum Elliott	E	100,9	28,6	2	10
Polygonum sp.	E	86,1	0	7	0
Polygonum stelligerum Cham.	E	30,4	0	2	0
Pontederia rotundifolia L. f.	RFL	29,4	52,2	3	2
Potamogeton pusillus L.	RS	2,6	3,4	2	3
Ricciocarpus natans	FF	0,0	0,1	6	9
Sagittaria montevidensis Cham. & Schltdl. subsp. *montevidensis*	E	22,1	3,7	6	2
Salvinia spp.	FF	30,0	21,1	19	15
Schoenoplectus californicus (C.A. Mey.) Soják var. *californicus*	E	53,6	0	1	0
Setaria parviflora (Poir.) Kerguélen var. *parviflora*	E	10,4	0	2	0
Soliva anthemifolia (Juss.) R. Br. ex Less.	E	0,6	0	1	0
Spirodela intermedia W. Koch	FF	0,1	0,1	4	4
Thalia geniculata L.	E	28,9	0	3	0
Utricularia foliosa L.	FS	0,0	1,3	0	10
Utricularia gibba L.	FS	0,9	1,3	6	3
Victoria cruziana Orb.	RFL	78,6	0	3	0
Wolffiella sp.	FF	0,1	0,2	5	2
