# Supplementary material for: Associations between Macrophyte Life Forms and Environmental and Morphometric Factors in a Large Sub-tropical Floodplain
Source: Front Plant Sci. 2018 Feb 19;9:195. doi: 10.3389/fpls.2018.00195 (PMC5826054; doi:10.3389/fpls.2018.00195)
Supplement: Supplementary file 4 [file SupplementaryMaterial4.pdf]

Supplementary Material 4

Article — **Associations between macrophyte life forms and environmental and morphometric factors in a large sub-tropical floodplain**

List of Authors — Berenice Schneider*, Eduardo Ribeiro Cunha, Mercedes Marchese and Sidinei Magela Thomaz

***Correspondence:** Berenice Schneider: bereschneider@gmail.com

Supplementary Material 4 Results of redundancy analysis (RDA) performed to assess relationships of biomass composition of macrophyte functional groups and environmental variables. Environmental variables are degree of connectivity (Conec), depth, hydrological period (Hyd), conductivity (Cond) and nitrate (Nit).

RDA axis	Proportion explained	Standardized regression coefficient				
		Conec	Depth	Hyd	Cond	Nit
1	0.52	-0.154	0.092	-0.065	0.038	0.008
2	0.31	-0.028	-0.163	0.095	-0.087	0.120
3	0.13	0.029	-0.052	0.051	-0.190	0.013
4	0.04	0.000	-0.095	-0.109	-0.055	0.035
6	0.01	0.033	0.103	-0.123	0.026	0.132
